# Is Childhood Maltreatment Associated with Body Image Disturbances in Adulthood? A Systematic Review and Meta-Analysis

**DOI:** 10.1007/s40653-021-00379-5

**Published:** 2021-08-07

**Authors:** Christine Bödicker, Jonas Reinckens, Michael Höfler, Jürgen Hoyer

**Affiliations:** Institute for Clinical Psychology and Psychotherapy, Technical University of Dresden, Dresden, Germany

**Keywords:** Body image, Body dissatisfaction, Childhood maltreatment, Abuse, PTSD

## Abstract

**Purpose:**

We aimed to synthesize the evidence for an association between childhood maltreatment and body image disturbances in adulthood. Information on maltreatment subtypes and mediator variables was included to gain further insights into the mechanisms of the association. In addition, we aimed to examine the role of body image disturbances in the development of negative mental health outcomes associated with childhood maltreatment.

**Methods:**

Based on a comprehensive search strategy, eligible studies were identified in PubMed, Scopus, and Web of Science. The eligibility assessment was performed by two reviewers, and 132 articles were studied full-text. To reduce heterogeneity, only non-clinical samples were included in the meta-analysis. A meta-regression was computed to examine the influence of maltreatment subtype on body image disturbances.

**Results:**

Our results provide evidence for a robust association between childhood maltreatment and cognitive-affective body image, both in clinical and community samples. Included studies (*N* = 40) indicate that body image disturbances are especially pronounced in individuals suffering from Posttraumatic Stress Disorder (PTSD) after childhood maltreatment. The meta-analysis included 12 studies with a total of 15.481 participants, and indicates a small overall effect size (r = 0.21, 95% CI = [0.16, 0.26], *p* < .001). Meta-regression revealed no significant impact of maltreatment subtype in non-clinical samples.

**Conclusion:**

Childhood maltreatment should be considered as a distal risk factor for the development of a negative cognitive-affective body image. We argue for future longitudinal studies which allow a better understanding of the pathways linking childhood maltreatment, body image disturbances and associated psychopathology.

**Supplementary Information:**

The online version contains supplementary material available at 10.1007/s40653-021-00379-5.

## Introduction

A positive body image is a crucial factor for psychosocial functioning and subjective well-being (Cash & Fleming, 2002). Seeing the own body in a positive light has been described as essential for engagement in self-care behaviors (such as physical exercise or the use of sun protection; Andrew et al., 2016; Avalos et al., 2005), self-esteem (Williams et al., 2004), interpersonal confidence and social support (Thompson et al., 1999) as well as for sexual functioning (Gillen & Markey, 2019; Satinsky et al., 2012).

Conversely, a negative body image has been linked to poorer psychological adjustment and quality of life (Annunziata et al., 2012; Bullen et al., 2012; Cash & Fleming, 2002; Cash et al., 2004b). In non-clinical samples, body image disturbances and body-related shame have been found to be associated with negative health behaviors such as smoking and binge-drinking (King et al., 2005; Nelson et al., 2009), avoidance of health care services such as cancer screening (due to felt discomfort when urged to expose the body for examination) and lack of exercise (Mensinger et al., 2018; More et al., 2019; Ridolfi & Crowther, 2013).

These findings indicate the integral role of body image in mental and physical health. Nevertheless, methodological problems presently prevent a deeper understanding of the etiopathogenetic pathways that may result from a negative body image.

A major challenge in body image research is the broad conceptualization of the construct (Thompson, 2004; Thompson et al., 2012). In the literature, body image is referred to a variety of terms (often reducing the construct to one of its components) such as body esteem, body schema, body concern, appearance evaluation, appearance orientation, size perception accuracy, body satisfaction, weight satisfaction, and drive for muscularity or thinness (Grogan, 2010; Thompson et al., 1999). As conceptualized by Slade (1994), body image is more complex and comprises the mental representation of body size and shape, body-related feelings and behaviors. Body image development is thought to be influenced by biological as well as cultural factors (Slade, 1994; Slevec & Tiggemann, 2011). Reflecting the multidimensionality of the construct, it is commonly differentiated between perceptive, cognitive-affective and behavioral components of body image (Thompson et al., 1999). The perceptive component refers to the estimation of actual body size and shape. The cognitive-affective component consists of two subcomponents: the importance attributed to weight and shape and the appraisal of one’s own appearance (Cash et al., 2004a, b). A negative self-evaluation of the own body is referred to as body dissatisfaction (Cash & Pruzinsky, 1990). Control strategies (e.g., restricted eating) and avoidance behavior (e.g., wearing loose instead of tight-fitting clothing) are subsumed under the behavioral component. In this review, we focus on the cognitive-affective component of body image.

Due to the harmful impact of a negative body image on mental and physical health, a growing body of research focuses on risk factors for the development of body dissatisfaction. Internalization of beauty ideals and appearance-related social comparison (Carlson Jones, 2004; Fuller-Tyszkiewicz et al., 2019), self-objectification (Augustus-Horvath & Tylka, 2009; Slevec & Tiggemann, 2011), exposure to idealized media images and social media use (Fardouly & Vartanian, 2016; Hargreaves & Tiggemann, 2004), low self-esteem and weight-related teasing (Ata et al., 2007; Chen et al., 2007; Valois et al., 2019), heightened body mass (Barker & Galambos, 2003; Calzo et al., 2012), and deficits in social support (Gerner & Wilson, 2005; Stice & Whitenton, 2002) have been identified as risk factors.

Early on, observations of clinicians indicated that a history of childhood sexual abuse also constitutes a risk factor for the development of body image disturbances (Arvanitakis et al., 1993; Myers, 1989; Simonds, 1992). In view of a developmental perspective, it is plausible that severe violations of body boundaries in a sensitive period for the embodiment of personal identity and integrity like those caused by several subtypes of childhood maltreatment have persisting effects on the perception of the self and the own body (Arvanitakis et al., 1993; Kearney-Cooke & Striegel-Moore, 1994; Knafo, 2016; Young, 1992).

Childhood maltreatment is commonly defined as emotional, physical and sexual abuse, and emotional and physical neglect of a minor younger than 18 years by an adult or an older adolescent with pronounced maturational difference.

Any sexual contact with a child under the age of 18 years by an authority or care-taker, and sexual acts forced by violence or trickery have been defined as childhood sexual abuse (CSA) by Finkelhor (1984). Physical abuse (CPA) is commonly described as harmful acts towards a child such as overt violence and excessive punishment (including exposure to extreme temperature or poisoning), resulting in injury or risk of injury (Kelly, 1983; Malinosky-Rummell & Hansen, 1993). Emotional abuse (CEA) can be defined as adverse parental behavior leading to impairments in the child’s psychological functioning and emotional well-being such as verbal assaults, harsh criticism, rejection and ignoring (Bernstein et al., 2003; Thompson & Kaplan, 1996). Whereas the different types of abuse are characterized by harmful actions, neglect is defined as the omission of behavior essential for the child’s healthy development (Mennen et al., 2010). Physical neglect (CPN) constitutes a lack of parental supervision which puts the child in danger, and a failure to meet basic needs by providing food, health care, and shelter (Bernstein et al., 2003). Emotional neglect (CEN) has proved to be especially difficult to define and can be understood as emotional unresponsiveness and unavailability of the care-taker violating the child’s basic need for emotional nurturance (Glaser, 2011). Different kinds of childhood maltreatment frequently co-occur (Dong et al., 2004).

Considering the overlap as well as the specific characteristics of the five childhood maltreatment subtypes, the question arises whether initial clinical observations of body image disturbances in victims of sexual abuse can be generalized to all kind of maltreatment experiences.

**Fig. 1:**
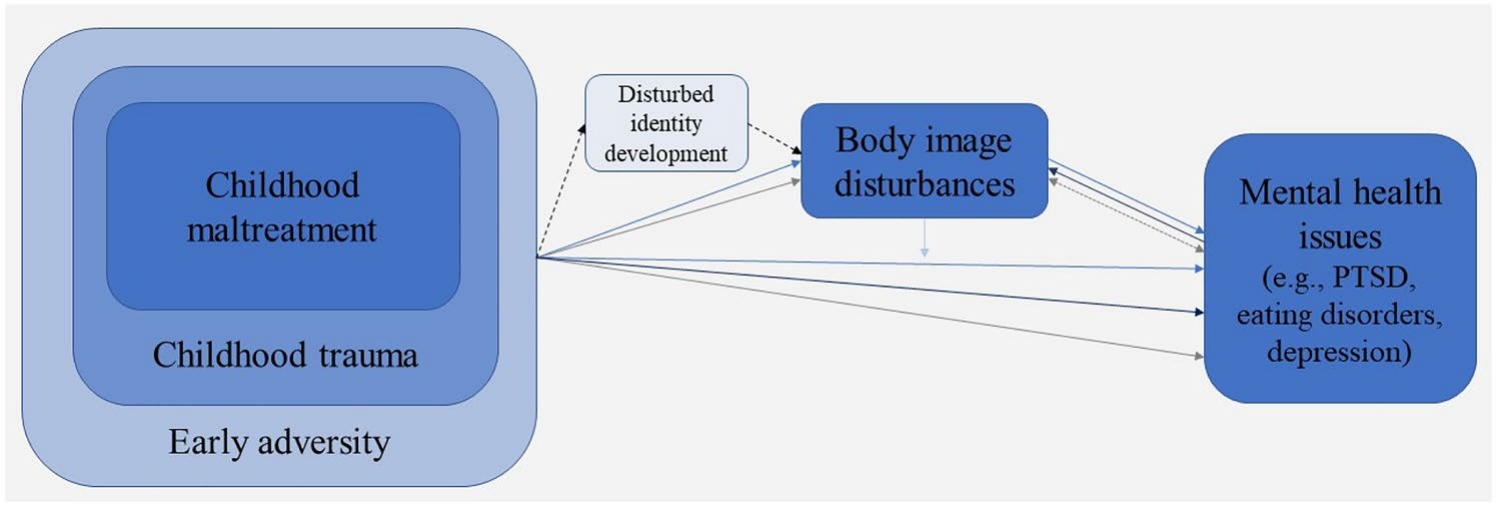
Theoretical considerations on the association between childhood maltreatment, body image and mental health issues. Different colors depict different potential mechanisms (mediation, moderation, simple association) linking the constructs. The path between CM and body image is assumed to be more complex, for a detailed description of the Identity disruption model please see Vartanian et al. (2018)

In the identity disruption model, Vartanian et al. (2018) focus on early adversity as a broad construct including general negative experiences and childhood trauma. According to this conceptualization, early adversity includes experiences such as growing up in an unstable family environment as well as experiences of abuse and neglect (as depicted in Fig. 1). Vartanian et al. (2018) assume that such early adversity disturbs normal identity development, and that individuals who lack well-formed personal identity and self-concept clarity are especially vulnerable to sociocultural influences as they look for external sources in order to attain a stronger sense of self. Hence, individuals with experiences of early adversities are supposed to be especially vulnerable to sociocultural pressures like beauty standards and appearance feedback, internalization of the latter and engagement in social comparisons regarding their body. Internalization of beauty ideals and engagement in body-related social comparisons are thought to be interrelated and to lead to increased body dissatisfaction. According to the identity disruption model (Vartanian et al., 2018), body dissatisfaction is associated with restrained eating, bingeing, purging and compulsive exercise.

In empirical studies, body image disturbances following childhood maltreatment were observed not only in eating disorder patients (e.g., Grilo & Masheb, 2001; Rayworth et al., 2004), but in a variety of physical and mental health conditions such as PTSD (Dyer et al., 2015; Scheffers et al., 2017), borderline personality disorder (BPD; Dyer et al., 2013a, b; Haaf et al., 2001), major depressive disorder (Andrews, 1995; Csoboth et al., 2005), poor sexual functioning (Wenninger & Heiman, 1998), and obesity (Duncan et al., 2015).

To enhance the clinical benefits of these observations and to prevent the development of body image disturbances, it is crucial to understand the pathways linking childhood maltreatment, body image disturbances and mental health issues. As depicted in Fig. 1, body image may partially mediate (or in some cases even moderate) the association between childhood maltreatment and psychopathology, could be understood as associated symptom of specific disorders, or the association between body image and mental health could be observed simply because both constructs are negatively influenced by experiences of childhood maltreatment.

Evidence for body image alterations in individuals with a maltreatment history in non-clinical samples (e.g., Hunter, 1991; Kearney-Cooke & Ackard, 2000) supports the assumption that experiences of childhood maltreatment disturb body image development (independent of subsequent psychopathology).

In conclusion, childhood maltreatment occurs in a sensitive developmental period for the embodiment of personal identity and is supposed to disturb the latter (Krueger, 2002; Young, 1992). Individuals who lack well-formed personal identity might be especially vulnerable to sociocultural influences like beauty ideals and thus tend to develop body image disturbances (Vartanian et al., 2018). Whereas some childhood maltreatment subtypes affect the body directly (by causing pain and injury), others more prominently affect psychological functions such as the self-image and self-esteem. In empirical studies, body image alterations have been documented to occur after childhood maltreatment both in clinical and population samples. However, a systematic review of the evidence for the association between childhood maltreatment and cognitive-affective body image in adults is lacking.

The aims of this article are twofold. First, we will synthesize the present empirical evidence for the association between childhood maltreatment and cognitive-affective body image in clinical and community samples. This part of the review will especially try to answer the question whether all subtypes of childhood maltreatment are comparable in terms of their association with cognitive-affective body image or whether certain subtypes of childhood maltreatment must be considered specifically relevant in this regard. Secondly, we will analyze evidence for the potential etiopathogenetic pathways through which body image disturbances might develop and function after abuse, taking into account the methodological quality of the underlying studies. More specifically, we try to answer the question whether body image disturbances should be considered a causal factor, moderator, mediator or simply an associated symptom in pathogenetic models of the sequelae of childhood maltreatment.

## Methods

This review and the including meta-analysis were performed according to the Preferred Reporting Items for Systematic Reviews and Meta-Analyses (PRISMA) statement (Moher et al., 2009).

### Data Sources

PubMed, Scopus, and Web of Science were searched for articles on childhood maltreatment (CM) and body image (allowing for results on associated keywords like body dissatisfaction, body esteem and bodily shame). Data bases were chosen based on relevance and coverage (Halladay et al., 2015; Visser et al., 2021). In addition, references of included studies were revised for further studies meeting the inclusion criteria.

### Search Process

The data-base driven research was conducted in May and June 2019, the search term ("body image" OR "body dissatisfaction" OR "body esteem" OR "bod* shame" OR "drive for thinness" OR "drive for muscularity") AND ("child* maltreatment" OR "child* abuse") was applied. An update literature search was performed from April to May 5th 2020.

### Inclusion and Exclusion Criteria

Quantitative studies on the association between cognitive-affective body image and childhood maltreatment (defined as sexual, physical or emotional abuse and emotional or physical neglect before the age of 18 years) in adults published in English between January 1990 and May 2020 were included in this review. Studies on lifetime sexual or physical abuse (including traumatic experiences in childhood and adulthood with no differentiation) were not included. Given the scarcity of research on this topic, studies primarily focusing on different outcomes of CM that also covered measures of body image (including body shame) were included as well.

### Eligibility Assessment

Out of 390 identified records, 131 articles were selected based on title and abstract. One additional article was selected from the reference list of one of the included articles. In the next step, the 132 selected articles were studied full-text in order to evaluate study quality and application of inclusion criteria. Each step of the eligibility assessment (screening of title and abstract, full-text analysis) was performed by two reviewers, discrepancies were resolved using consensus.

### Data Extraction

Information was extracted from each included study on: (1) sample characteristics (including age, gender, diagnosis, type of childhood maltreatment and measure of the latter), (2) type of outcome measure (operationalization of body image) and (3) results of statistical analyses on the association between childhood maltreatment and body image (including further mediator or moderator variables).

### Study Quality and Risk of Bias

Study quality and risk of bias were assessed independently by two reviewers applying the NIH Quality Assessment for Observational Cohort and Cross-sectional studies (National Heart Lung and Blood Institute, 2018). Discrepancies were resolved by consensus. Out of the 14 items of the NIH checklist, 9 items were applicable for the purpose of this review. Items regarding research question, sample description, participation rate, target population, sample size justification, measurement of exposure (CM) and outcome (body image), and inclusion of confounding variables were answered with yes, no, cannot determine (CD), not reported (NR) or not applicable (NA). Based on the literature, we predefined gender, age, and BMI as important confounding variables. Studies including various categories of CM received higher ratings on item 8 compared to studies focusing on one type (e.g., childhood sexual abuse). If customized measures of CM (without evidence for validity and reliability) were applied we rated item 9 as CD. Given the theoretical range of 0–9 points, we rated 7–9 points (> 80%) as good, 5,5–7 points (60–80%) as fair and less than 5,5 points (< 60%) as poor quality. This categorization is comparable with the approach of van Dalen et al. (2020).

#### Meta-analysis

In order to reduce heterogeneity, only studies including non-clinical samples and body dissatisfaction, body esteem or shape concerns as outcome variable were included in the meta-analysis. If data necessary for computation were missing, first authors were contacted and asked to provide additional data within four weeks (except for one study, for which author contact details could not be obtained). A total of 8 authors were contacted and response rate was 62.5%. In case of multiple assessments of body image in one study, only one result was entered into the analysis. Thereby, we focused on body dissatisfaction and shape concerns as outcome.

If necessary, effect sizes were converted based on the recommendations of the Campbell Methods Policy Note on Converting Between Effect Sizes (Polanin & Snilstveit, 2016) using the Practical Meta-Analysis Effect Size Calculator (Wilson, 2016). Publication bias was assessed by visual inspection of a funnel plot, Begg’s rank test (Begg & Mazumdar, 1994) and Egger’s regression (Egger et al., 1997). As I^2^-statistic indicated considerable heterogeneity with I^2^ = 71.7% (Melsen et al., 2014), a random-effects model was chosen. To assess whether the CM subtype influenced the association between CM and body dissatisfaction, a meta-regression with CM subtype as independent variable was computed in Stata version 15.1 by the “metareg” command (StataCorp, 2015). Due to differing assessment of CM in the included studies, 8 categories were entered in the analysis (1 = CEA, 2 = CPA, 3 = CSA, 4 = CEN, 5 = CPN, 6 = CM, 7 = CPA + CSA, 8 = neglect).

## Results

### Systematic Review

**Fig. 2:**
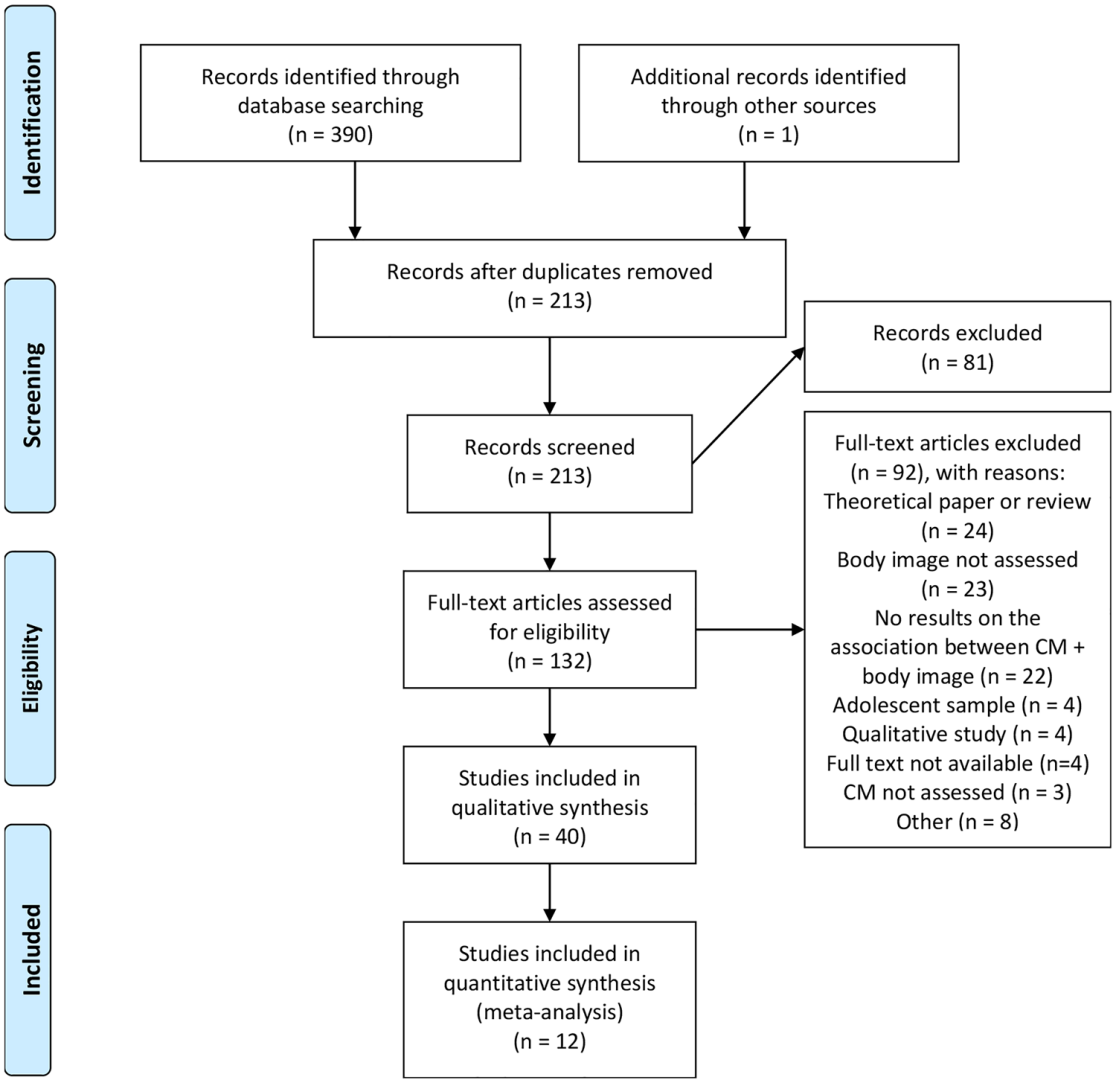
PRISMA Flow Diagram: Search and selection process

In total, 40 studies have been included in this review. 37 studies provide evidence for a significant association between childhood maltreatment and cognitive-affective body image and adjacent constructs such as body shame (six studies) and body esteem (four studies). The eligibility assessment is depicted in Fig. 2.

### Sample Characteristics

19 of the included studies provide information on the association between childhood maltreatment and body image in non-clinical samples. These studies include community and university samples, but also focus on specific target populations such as pregnant women (Senior et al., 2005; Talmon & Ginzburg, 2019), prisoners (Milligan & Andrews, 2005), individuals with migration history (Nagaraj et al., 2019) and intimate piercings (Möller et al., 2018). 20 studies included clinical samples such as obese individuals (Grilo et al., 2006, 2005a, b; Rohde et al., 2008; Walsh et al., 2017), women diagnosed with sexual dysfunction (Kilimnik & Meston, 2016; Maseroli et al., 2018) or breast cancer (Salmon et al., 2006). In regard to mental health conditions associated with childhood maltreatment and body image disturbances, the reviewed studies mostly focus on PTSD or patients in trauma-related treatment (Borgmann et al., 2014; Dyer et al., 2015, 2013a, b; Scheffers et al., 2017; Wonderlich et al., 1996, 2001), eating disorders (Dunkley et al., 2010; Grilo & Masheb, 2001; Muehlenkamp et al., 2011; Treuer et al., 2005), and BPD (Dyer et al., 2015, 2013a, b). One study included a mixed psychiatric sample (Zlotnick et al., 1996). Bandini et al. (2011) studied individuals with male-to-female gender identity disorder (MtF GID).

In 27 of the included studies (including one study based on individuals with MtF GID), the association between childhood maltreatment and body image disturbances has been investigated in women only, and in ten of the remaining studies a balanced gender ratio could not be attained (resulting in a higher proportion of women). The three studies including balanced gender ratios point towards potential gender specific effects of CM on body image. The results of Brooke and Mussap (2013) indicate that abused men tend to develop a strong drive for thinness whereas abused women show a heightened drive for muscularity.

Please see Table S1 in the supplementary material for an overview of maltreatment subtype, body image measures, and sample characteristics of the included studies.

### CM Subtypes

Multiple types of CM were assessed in 57.5% of the included studies. In the remaining studies, only one type of CM was assessed: 16 studies (40%) focused on CSA (including one study assessing incest only; Wonderlich et al., 1996) and one study assessed CEA only (Hund & Espelage, 2006). Overall, CSA was assessed most frequent in the included studies, followed by CPA and CEA and a comparatively low rate of the assessment of emotional and physical neglect (27.5%). The qualitative results indicate differing strength of association between childhood maltreatment subtypes and cognitive-affective body image.

### Measures

As depicted in Table S1 in the supplementary material, included studies differ in sample size and applied measures. Whereas childhood maltreatment has been assessed mainly by the Childhood Trauma Questionnaire (CTQ; Bernstein & Fink, 1998), measures and operationalization of body image differ. Most frequently, the subscales body dissatisfaction and drive for thinness of The Eating Disorder Inventory (EDI; Garner et al., 1983), the Body Shape Questionnaire (BSQ; Cooper et al., 1987) and the subscales shape and weight concerns of the Eating Disorder Examination Questionnaire (EDE-Q; Fairburn & Beglin, 1994) have been chosen to assess aspects of cognitive-affective body image.

### Study Design

In the majority of the included studies (92.5%), a cross-sectional study design was applied. Only three studies included longitudinal data. Grilo et al. (2006) compared pre- and 12 months post-operation outcomes in gastric bypass patients. Senior et al. (2005) included data from measurements at 18 and 32 weeks’ gestation to assess the influence of early traumatic experiences on maternal eating disorder symptoms. Andrews (1995) used longitudinal data to study the association between childhood maltreatment, body shame and depression.

### Study Quality and Risk of Bias

The mean quality score of the included studies was 5.9 (range 3.5 – 8), which indicates a fair overall study quality. Common minor flaws were missing sample size justification, participation rate, and no specification of time period and place of recruitment. Overall, seven studies were considered of good quality, 22 of fair quality, and eleven of poor quality. The quality assessment is shown in Table S2 in the supplementary material.

### Body Image as Mediator Variable

Initial support for the identity disruption model by Vartanian et al. (2018) was obtained. According to the model, early adversity leads to impaired self-concept clarity which makes individuals vulnerable to the internalization of sociocultural influences and the engagement in body comparison. This liability is assumed to lead to body dissatisfaction and associated disordered eating patterns (Vartanian et al., 2018). These assumptions are in line with the results of Preti et al. (2006), who identified body dissatisfaction as a mediator in the association between CSA and eating disorder symptoms.

According to Rohde et al. (2008) body dissatisfaction does not mediate the association between childhood maltreatment and adult obesity in middle-aged women.

Muehlenkamp et al. (2011) report an indirect effect of CM on non-suicidal self-injury (NSSI) via body dissatisfaction, low self-esteem, psychopathology, and dissociation.

Andrews (1995) found bodily shame to be a mediator between childhood maltreatment and chronic depression.

Talmon and Ginzburg (2019) suppose that the association between CM and fear of childbirth is partially mediated by self-objectification, disruption in body boundaries and body shame.

### The Impact of Posttraumatic Stress Disorder (PTSD)

PTSD patients with a history of childhood maltreatment show pronounced alterations in body image, including body-related feelings of shame and disgust (Borgmann et al., 2014; Dyer et al., 2015, 2013a, b; Scheffers et al., 2017; Walsh et al., 2017). Notably, body-related emotions and impaired body experience were shown to correlate with PTSD severity, but not with trauma severity and dissociation within the PTSD group (Borgmann et al., 2014; Scheffers et al., 2017). Dyer et al. (2015) report more negative body-related feelings in PTSD patients compared to BPD patients (both with a history of CSA). According to Walsh et al. (2017), abuse survivors (CPA + CSA) suffering from PTSD report less body satisfaction compared to abuse survivors without PTSD (*p* = 0.015).

### Meta-analysis

#### Study Characteristics

12 studies were included in the meta-analysis. These studies included only non-clinical samples and 15.481 participants in total, of which 95% were female. Sample size ranged from 38 to 7806, with a medium sample size of 1.290. Mean age of participants was 34.5 years with a range from 18 to 64 years. Study characteristics are described in Table 1. Please note that sample sizes mentioned above describe the sample size for which relevant data in regard to the present research question were available, whereas Table 1 depicts overall sample sizes.

**Table 1:** Studies included in the meta-analysis

author	sample characteristics	*N*	gender (female)	CM type (measures)	body image measures
Andrews, 1995	working-class mothers	84	f	CPA + CSA (semi-structural interview)	numerical ratings based on interview
Brooke & Mussap, 2013	community sample	299	b (52%)	CEA, CPA, CSA, CEN + CPN (CTQ)	EDE-Q, EDI, DMS
Eubanks et al., 2006	college students	38	f	CPA + CSA (CHQ)	BESAA
Hund & Espelage, 2006	university students	608	f	CEA (CTQ; CATS)	EDI; EAT-26
Jenkins et al., 2013	university students	118	f	CSA, CPA, CEA and neglect (CATS)	EDI-2
Mahtani et al., 2019	nonclinical sample, with NSSI (*220*)	573	b (69%)	CEA, CSA, CPA, CPN (4 items adapted from CTQ)	BES
Möller et al., 2018	individuals with intimate piercings	72	b (66%)	CEA, CPA, CSA, CEN + CPN (CTQ)	FBeK
Preti et al., 2006	community sample	126	f	CSA (customized questions)	BAT
Rohde et al., 2008	middle-aged community sample stratified based on high BMI	4,641	f	CPA + CSA (interview questions adapted from CTQ)	single item
Senior et al., 2005	pregnant women	10,641	f	CEA, CPA, CSA (customized questionnaire)	EDE-Q
Vartanian et al., 2018	young adults (18–30 years)	1,023	b (52%)	CEA, CPA, CSA + CPN (CTQ, RFQ)	EDE-Q
Wenninger & Heiman, 1998	community sample	104	f	CSA (telephone interview)	BES, MBSRQ

*BAT* The Body Attitude Test (Probst et al., 1995), *BES* The Body Esteem Scale (Franzoi & Shields, 1984), *BESAA* Body-esteem scale for adolescents and adults (Mendelson & White, 1982)*, CATS* The Child Abuse and Trauma Scale (Sanders & Becker-Lausen, 1995), *CEA* childhood emotional abuse, *CHQ* Childhood History Questionnaire (Milner et al., 1990), *CPA* childhood physical abuse, *CSA* childhood sexual abuse, *CTQ* Childhood Trauma Questionnaire (Bernstein & Fink, 1998), *DMS* Drive for Muscularity Scale (McCreary, 2007), *EAT-26* Eating Attitudes Test-26 (Garner et al., 1982), *EDE-Q* Eating Disorder Examination Questionnaire (Fairburn & Beglin, 1994), *EDI* The Eating Disorder Inventory (Garner, 2004), *EDI-2* The Eating Disorder Inventory–2 (Garner, 1991), *FbeK* Fragebogen zur Beurteilung des eigenen Körpers *[Questionnaire for evaluations of one’s own body*] (Strauß & Richter-Appelt, 1996), *HC* healthy controls, *MBSRQ* Multidimensional Body-Self Relations Questionnaire (Brown et al., 1990), *NSSI* non-suicidal self-injury, *RFQ* Risky Family Questionnaire (Taylor et al., 2004)

#### Study Quality and Risk of Bias

The mean quality score of the included studies was 6 (range 3.5 – 8), which indicates a fair overall study quality. Overall, two studies were considered of good quality, eight of fair quality, and two of poor quality. The quality assessment is shown in Table 2.

**Table 2:** Quality Assessment of studies included in the meta-analysis

	1.Research Question	2.Study population	3.Partici-pation rate	4.Recruit-ment	5. Power	6.Catego-ries of exposure	7.Expo-sure measures	8.Out-come measures	9.Con-founders	Total scores	Rating
Andrews (1995)	1	0,5	NR	1	0	0	CD	0,5	0,5	3,5	poor
Brooke and Mussap (2013)	1	1	NA	1	0	1	1	1	1	7	good
Eubanks et al. (2006)	1	1	NA	1	0	0,5	1	1	0,5	6	fair
Hund and Espelage (2006)	1	1	NA	1	0	0,5	1	1	1	6,5	fair
Jenkins et al. (2013)	1	1	NA	1	0	1	1	1	0,5	6,5	fair
Mahtani et al. (2019)	1	1	NA	1	0,5	1	0,5	1	0,5	6,5	fair
Möller et al. (2018)	1	1	NR	1	0	1	1	1	0	6	fair
Preti et al. (2006)	1	0,5	0,5	1	0,5	0	0	1	1	5,5	fair
Rohde et al. (2008)	1	1	1	1	0,5	0,5	0	0	0,5	5,5	fair
Senior et al. (2005)	1	1	NR	1	0	0,5	CD	0,5	0,5	4,5	poor
Vartanian et al. (2018)	1	1	1	1	0	1	1	1	1	8	good
Wenninger and Heiman (1998)	1	1	1	1	0	0,5	CD	1	1	6,5	fair

1. Was the research question or objective in this paper clearly stated? 2. Was the study population clearly specified and defined? 3. Was the participation rate of eligible persons at least 50%? 4. Were all subjects selected or recruited from the same or similar populations? Were inclusion and exclusion criteria for being in the study prespecified and applied uniformly to all participants? 5. Was a sample size justification, power description, or variance and effect estimates provided? 6. For exposures that can vary in amount or level, did the study examine different levels of the exposure as related to the outcome (e.g., categories of exposure, or exposure measured as continuous variable)? 7. Were the exposure measures (independent variables) clearly defined, valid, reliable, and implemented consistently across all study participants? 8. Were the exposure outcome measures clearly defined, valid, reliable and implemented consistently across all study participants? 9. Were key potential confounding variables measured and adjusted statistically for their impact on the relationship between exposure and outcome? Given the theoretical range of 0–9 points, we rated 7–9 points (> 80%) as good, 5,5–7 points (60–80%) as fair and less than 5,5 points (< 60%) as poor quality

*CD* Cannot Determine, *NA* Not Applicable, *NR* Not Reported

#### Overall Effect Size

**Fig. 3:**
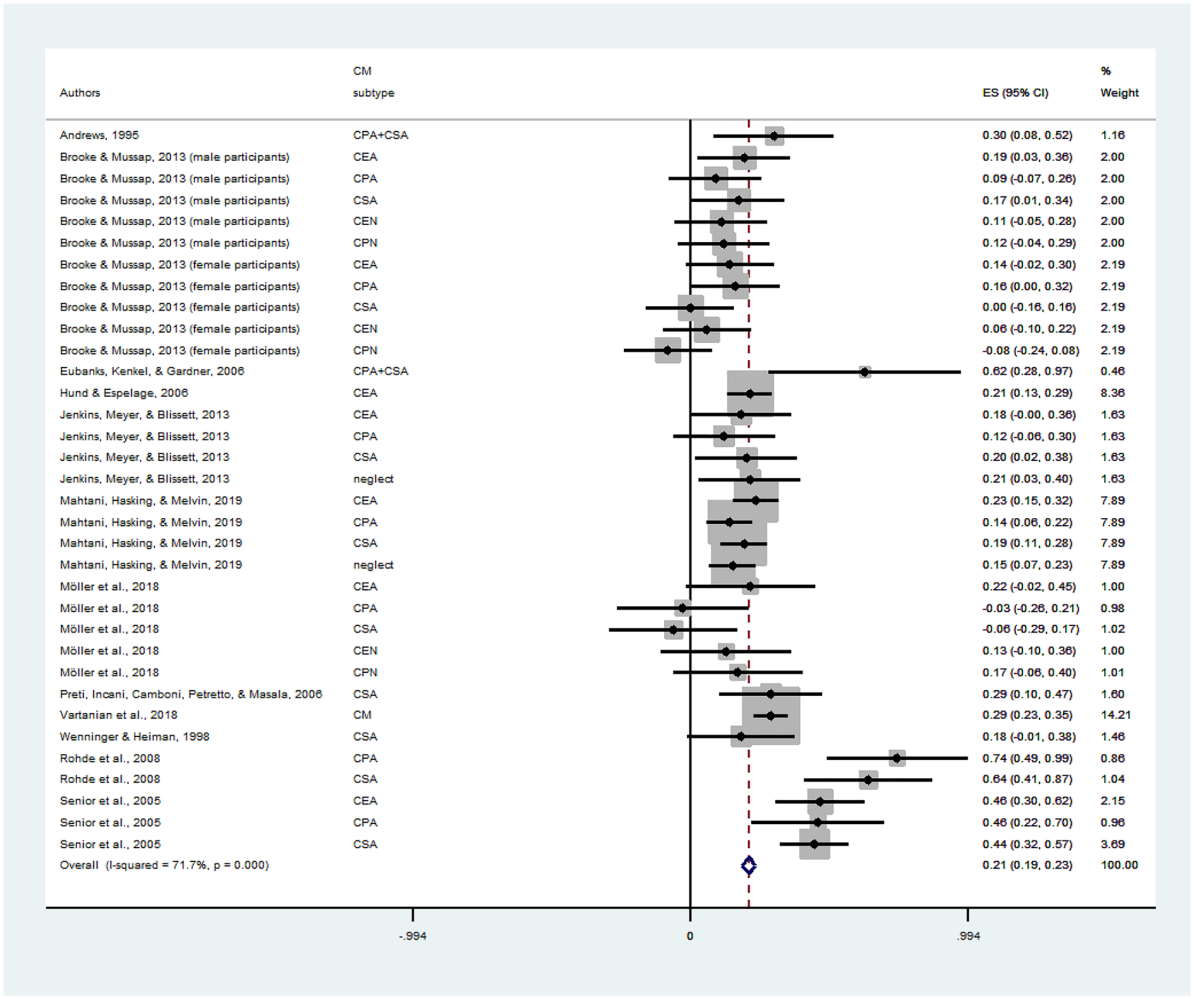
Forest plot: Results of the meta-analysis

The results of the meta-analysis are depicted in Fig. 3. Results show a small overall effect size for the association between childhood maltreatment and body image (to be more precise: body esteem, body dissatisfaction or shape concerns) across included studies (r = 0.21, 95% CI = [0.16, 0.26], *p* < 0.001). As noted above, a high level of heterogeneity was observed (I^2^ = 71.7%, *p* < 0.001). The funnel plot was symmetric. Egger’s regression (*p* = 0.948) and Begg’s rank test (*p* = 0.166) were non-significant and indicated the absence of publication bias. Meta-regression indicated that CM subtype did not influence the association between CM and body dissatisfaction with *F*(7, 28) = 0.92, *p* = 0.5068. Sensitivity analyses were conducted by excluding OR studies (Rohde et al., 2008; Senior et al., 2005), which changed the pattern and indicated an effect of CM subtype [*F*(7, 21) = 4.48, *p* = 0.0034].

## Discussion

We systematically reviewed the literature on the association between childhood maltreatment and body image disturbances in adults. We aimed to answer the research question whether childhood maltreatment subtypes differ in their impact on body image and to analyze evidence for the potential etiopathogenetic pathways through which body image disturbances might develop and function after abuse and neglect.

Our results underline the relevance of body image disturbances in individuals with a history of childhood maltreatment. Of 40 studies included in the systematical review, 37 studies provide evidence for a significant association between childhood maltreatment and cognitive-affective body image. Meta-analysis revealed a small association between childhood maltreatment and body-related attitudes and feelings in non-clinical samples (r = 0.21, 95% CI = [0.16, 0.26], *p* < 0.001). Results of the meta-regression should be interpreted with caution as data might be correlated due to the frequent co-occurrence of different maltreatment subtypes (Dong et al., 2004). The cross-sectional design of the included studies impedes conclusions about etiopathogenetic pathways, though single studies indicate that body image disturbances might mediate the association between childhood maltreatment and eating disorders (Preti et al., 2006; Vartanian et al., 2018) and that PTSD is associated with pronounced alterations in body image, including body-related feelings of shame and disgust (Borgmann et al., 2014; Dyer et al., 2015, 2013a, b; Scheffers et al., 2017; Walsh et al., 2017).

Studies included in this review vary widely in terms of body image operationalization and target population. We reviewed data of individuals with physical health conditions such as obesity (Grilo et al., 2006, 2005a, b; Rohde et al., 2008; Walsh et al., 2017), sexual dysfunction (Kilimnik & Meston, 2016; Maseroli et al., 2018) and breast cancer (Salmon et al., 2006), as well as of individuals with mental health conditions such as PTSD (Borgmann et al., 2014; Dyer et al., 2015, 2013a, b; Scheffers et al., 2017; Wonderlich et al., 1996, 2001), eating disorders (Dunkley et al., 2010; Grilo & Masheb, 2001; Muehlenkamp et al., 2011; Treuer et al., 2005), and BPD (Dyer et al., 2015, 2013a, b). The non-clinical samples included community samples, but also specific samples such as pregnant women (Senior et al., 2005; Talmon & Ginzburg, 2019), prisoners (Milligan & Andrews, 2005), and individuals with migration history (Nagaraj et al., 2019) or intimate piercings (Möller et al., 2018). Evidence drawn from such diverse target populations underlines the integral role of body image in mental and physical health, and indicates its relevance beyond the field of eating disorders. Childhood maltreatment should be considered as a distal risk factor for the development of a negative cognitive-affective body image, both in clinical and community samples.

To our knowledge, this is the first review of the association between childhood maltreatment and body image disturbances in adults.

Slevec and Tiggemann (2011) reviewed risk factors for body dissatisfaction and disordered eating in middle-aged women and concluded that sexual and physical abuse are consistently associated with disordered eating, and to a lesser degree with body dissatisfaction. Overall, they identified BMI and appearance-related teasing as main risk factors for the development of body dissatisfaction. Their results also underline the relevance of aging anxiety, self-objectification, perfectionism, self-esteem and to some extent sociocultural pressures for body dissatisfaction in middle-aged women.

Mostly, review articles in the field do not include childhood maltreatment but focus on other risk factors for the development of body dissatisfaction. Meta-analytical findings indicate small overall effect-sizes for thin media exposure in both genders, ranging from r = 0.03 to r = 0.17 (Ferguson, 2013; Grabe et al., 2008; Groesz et al., 2002; Levine & Murnen, 2009). Fat talk (r = 0.34), weight-related teasing (r = 0.39) and appearance-related teasing (r = 0.32) seem to be especially relevant to body dissatisfaction development in adults (Menzel et al., 2010; Mills & Fuller-Tyszkiewicz, 2017). Interestingly, the association between fat talk and later body dissatisfaction becomes trivial (r = 0.08) in longitudinal studies (Mills & Fuller-Tyszkiewicz, 2017).

Our meta-analytical findings are limited, as we only included non-clinical samples. However, the influence of childhood maltreatment on body dissatisfaction seems to be comparable to other known risk factors such as thin media exposure and is observed despite the time gap between maltreatment and body image assessment. We know little about intervening processes, but our results indicate that the link between childhood maltreatment and body image disturbances in adulthood may not be close and direct, but rather calls for a more differentiated view.

When interpreting our results, the complexity of prolonged trauma sequelae should be kept in mind. We suppose body image disturbances to be a meaningful yet slightly understudied factor, but only one among many to influence the association between childhood maltreatment and mental and physical health. Family environment, emotion regulation and impulsivity have been proven to play an important role in the development of negative mental and physical health conditions in individuals with a history of childhood maltreatment (Bhandari et al., 2011; Brown et al., 2017; Michopoulos et al., 2015). In addition, the influential role of neurobiological mechanisms like alterations in HPA axis functioning in the aftermath of early traumatic experiences remains unquestioned (Heim et al., 2008; Tarullo & Gunnar, 2006; Teicher et al., 2003).

During the conduction of this review and meta-analysis, we faced several challenges that might be familiar to researchers in the field of body image research. First, the multidimensionality of the construct and the variety of terms body image is referred to in the literature represent a challenge in the synthesizing of findings (Grogan, 2010; Thompson et al., 1999). Whereas we chose a broad approach for the systematic review by including adjacent constructs such as body shame, we focused on only one facet of body image in the meta-analysis in order to enhance comparability of results. While the association between body image disturbances and childhood maltreatment is robust when both constructs are considered in their broadest sense, the specificity of the association between diverse aspects of body image and subtypes of childhood maltreatment remains unclear and calls for a more fine-grained scientific analysis. Second, a gold standard for the assessment of body image is lacking and as research in this area has been growing in the last decade, so has the variety of instruments (Thompson, 2004; Thompson et al., 2012). For example, Kling et al. (2019) identified 150 different body images measures applied in recent years.

Synthesizing data from studies that vary strongly in sample characteristics, body image operationalization and assessment instruments is challenging and might limit the validity of our results. For future research, we support claims for a consensus on measurement choices in the field of body image research (Cash & Pruzinsky, 2002; Krawczyk et al., 2012).

It is also important to note that gender ratio was unbalanced and resulted in a higher proportion of women in the vast majority of reviewed studies. The results of the remaining studies indicate that the association between childhood maltreatment and body image might be gender-specific. Whereas our conclusions are mainly based on female samples, the inclusion of men would be desirable for future research on the topic.

Despite the afore-mentioned limitations, our results argue for the relevance of body image disturbances in those having undergone childhood maltreatment, a notion that has implications not only in terms of future research perspectives but also for clinical intervention. It is noteworthy that the majority of frequently applied evidence-based trauma therapies do not include work on body image – even though body image seems to be susceptible to therapeutic change (Farrell et al., 2006; Guest et al., 2019; Scheffers et al., 2017). Not surprisingly, body image alterations were identified among the residual symptoms of evidence-based, trauma-focused psychotherapies by Larsen et al. (2019). First results on the efficacy of body-related therapy adjacent to trauma-focused psychotherapy in women with a history of childhood sexual abuse are promising (Price, 2005). Body-related interventions seem to enhance the frequently reported low self-care of traumatized patients (Felitti et al., 1998); potentially leading to more health-conscious behaviors. Given the influential role of body image on quality of life and psychosocial functioning (Cash et al., 2004a, b; Scheffers et al., 2017), we suggest to include work on body image in therapeutic approaches focusing on maltreatment survivors.

## Supplementary Information

Below is the link to the electronic supplementary material.

Supplementary file1 (DOCX 58 KB)
