# Supplementary material for: Is Childhood Maltreatment Associated with Body Image Disturbances in Adulthood? A Systematic Review and Meta-Analysis
Source: J Child Adolesc Trauma. 2021 Aug 7;15(3):523–38. doi: 10.1007/s40653-021-00379-5 (PMC9360384; doi:10.1007/s40653-021-00379-5)
Supplement: Supplementary file 1 — Supplementary file1 (DOCX 58 KB) [file 40653_2021_379_MOESM1_ESM.docx]

Is childhood maltreatment associated with body image disturbances in adulthood?
A systematic review and meta-analysis

**Supplementary material**

Supplementary tables

Table S1

Studies included in the systematic review

| author | sample characteristics | *N* | gender (female) | CM type (measures) | body image measures |
| --- | --- | --- | --- | --- | --- |
| Andrews, 1995 | working-class mothers | 84 | f | CPA + CSA (semi-structural interview) | numerical ratings based on interview *(body shame)* |
| Bandini et al., 2011 | patients with MtF GID | 162 | MtF GID | CEA, CSA and neglect (interview) | BUT |
| Bhandari et al., 2011 | clinical + university sample | 106 | b (77 %) | CSA (customized questionnaire) | DSFI |
| Borgmann et al., 2014 | PTSD patients + HC | 46 | f | CSA (CTQ) | ER, TC *(body-related emotions and cognitions)* |
| Brooke & Mussap, 2012 | community sample | 299 | b (52 %) | CEA, CPA, CSA, CEN + CPN (CTQ) | EDE-Q, EDI, DMS |
| Dunkley, Masheb, & Grilo, 2010 | treatment-seeking overweight BED patients | 170 | b (78 %) | CEA, CPA, CSA (CTQ) | BSQ |
| Dyer et al., 2013 | BPD (*40),* BPD + lifetime eating disorders (*49*), HC (*41*) | 130 | f | CSA (CTQ) | BIAQ, MBSRQ |
| Dyer et al., 2013 | PTSD *(53),* PTSD + co-occurring eating disorders (*31*), HC (*53)* | 137 | f | CSA (CTQ) | DBII (DKB-35) + BIAQ |
| Dyer, Feldmann, & Borgmann, 2015 | PTSD (*23*), BPD (*25*), PTSD + BPD (*22*), HC (*27)* | 97 | f | CSA (CTQ) | BIGSS, SBA |
| Eubanks, Kenkel, & Gardner, 2006 | college students | 38 | f | CPA + CSA (CHQ) | BESAA *(body esteem)* |
| Grilo & Masheb, 2001 | BED outpatients | 145 | b (77 %) | CEA, CPA, CSA, CEN + CPN (CTQ) | BSQ |
| Grilo et al., 2005a | gastric bypass surgery candidates | 260 | b (83 %) | CEA, CPA, CSA, CEN + CPN (CTQ) | BSQ |
| Grilo et al., 2005b | gastric bypass surgery candidates | 340 | b (83 %) | CEA, CPA, CSA, CEN + CPN (CTQ) | BSQ |
| Grilo et al., 2006 | patients who underwent gastric bypass surgery | 137 | b (89 %) | CEA, CPA, CSA, CEN + CPN (CTQ) | EDE-Q; BSQ |
| Hund & Espelage, 2006 | university students | 608 | f | CEA (CTQ; CATS) | EDI; EAT-26 |
| Hunter, 1991 | nonclinical sample | 52 | b (54%) | CSA (interview, social history questionnaire) | BSRQ |
| Jenkins et al., 2013 | university students | 118 | f | CSA, CPA, CEA and neglect (CATS) | EDI-2 |
| Kilimnik & Meston, 2016 | patients with sexual dysfunction, distress, or low sexual satisfaction | 222 | f | CSA (CTQ, The Childhood Sexual Abuse Measure) | BES (*body esteem*) |
| Mahtani et al., 2019 | nonclinical sample, with NSSI (*220*) | 573 | b (69%) | CEA, CSA, CPA, CPN (4 items adapted from CTQ) | BES (*body esteem*) |
| Maseroli et al., 2018 | patients with sexual dysfunction | 200 | f | CSA (CECA.Q) | BUT |
| Milligan & Andrews, 2005 | prisoners | 89 | f | CPA + CSA (interview) | ESS (*body shame*) |
| Möller et al., 2018 | individuals with intimate piercings | 72 | b (66%) | CEA, CPA, CSA, CEN + CPN (CTQ) | FBeK |
| Muehlenkamp et al., 2011 | eating disorder patients | 422 | f | CEA, CPA, CSA (TEC) | BAT, EDI |
| Nagaraj, Vyas, & McDonnell, 2019 | South Asians living in the US | 535 | f | CEA, CPA, CSA (customized questions) | BESAA (*body esteem*) |
| Preti et al., 2006 | community sample | 126 | f | CSA (customized questions) | BAT |
| Rohde et al., 2008 | middle-aged community sample stratified based on high BMI | 4,641 | f | CPA + CSA (interview questions adapted from CTQ) | single item |
| Salmon et al., 2006 | breast cancer patients after surgery | 355 | f | CEA, CPA + CSA (customized questions) | ESS (*body shame*) |
| Schaaf & McCanne, 1994 | college students | 670 | f | CPA + CSA <13 years (CHQ) | EDI |
| Scheffers et al., 2017 | patients in treatment for early traumatization | 50 | f | CSA (patients’ reports in intake interview + during therapy ) | DBIQ-35-NL; BCS |
| Senior et al., 2005 | pregnant women | 10,641 | f | CEA, CPA, CSA (customized questionnaire) | EDE-Q |
| Talmon & Ginzburg, 2018 | nonclinical sample | 843 | b (64%) | CSA (CTQ) | OBC; ESS (*body shame*) |
| Talmon & Ginzburg, 2019 | pregnant women | 470 | f | CEA, CPA, CSA, CEN + CPN (CTQ) | ESS (*body shame*) |
| Treuer et al., 2005 | eating disorder patients | 63 | f | CPA + CSA (clinical interview) | BAT |
| Vartanian et al., 2018 | young adults (18-30 years) | 1,023 | b (52%) | CEA, CPA, CSA + CPN (CTQ, RFQ) | EDE-Q |
| Walsh et al., 2017 | bariatric surgery candidates | 3,045 | b (81%) | CPA + CSA (CTQ) | BASS |
| Watson et al., 2013 | university students | 538 | f | CSA (CTQ) | OBC (*body shame*) |
| Wenninger & Heiman, 1998 | community sample | 104 | f | CSA (telephone interview) | BES, MBSRQ |
| Wonderlich et al., 1996 | patients in treatment for incest | 65 | f | CSA, incest (interview) | EDI |
| Wonderlich et al., 2001 | patients in trauma-related treatment (*72) +* HC (*25*) | 97 | f | CSA (interview) | ASI, BIAQ, EDE |
| Zlotnick et al., 1996 | psychiatric sample | 134 | f | CSA (customized questionnaire) | EDI |

*Note.* ASI = Appearance Schemas Inventory (Cash & LaBarge, 1996), BASS = The Body Areas Satisfaction Scale (Multidimensional Body-Self Relations Questionnaire; Cash, Winstead, & Janda, 1986), BAT = The Body Attitude Test (Probst, Vandereyken, Van Coppenolle, & Vanderlinden, 1995), BCS = Body Cathexis Scale (Balogun, 1986; Secord & Jourard, 1953), BES = The Body Esteem Scale (Franzoi & Shields, 1984), BESAA = Body-esteem scale for adolescents and adults (Mendelson & White, 1982)*,* BIAQ = Body Image Avoidance Questionnaire (Rosen, Srebnik, & Saltzberg, 1991), BIGSS = Body image guilt and shame scale (Thompson, Dinnel, & Dill, 2003), BSS = The Body Satisfaction Scale (Slade, Dewey, Newton, Brodie, & Kiemle, 1990), BUT = Body Uneasiness Test (Cuzzolaro, Vetrone, Marano, & Battacchi, 1999), BSQ = The Body Shape Questionnaire (Cooper, Taylor, Cooper, & Fairburn, 1987), CATS = The Child Abuse and Trauma Scale (Sanders & Becker-Lausen, 1995), CECA.Q = The childhood experience of care and abuse questionnaire (Bifulco et al., 2005), CEA = childhood emotional abuse, CHQ = Childhood History Questionnaire (Milner, Robertson, & Rogers, 1990), CPA = childhood physical abuse, CSA = childhood sexual abuse, CTQ = Childhood Trauma Questionnaire (Bernstein & Fink, 1998), DBII (DKB-35) = Dresden Body Image Inventory (Dresdner Körperbildbogen; Pöhlmann, Thiel, & Joraschky, 2008), DBIQ-35-NL = Dutch version of the Dresden Body Image Questionnaire (Scheffers et al., 2016), DMS = Drive for Muscularity Scale (McCreary, 2007), DSFI = The Derogatis Sexual Functioning Inventory (Derogatis & Melisaratos, 1979), EAT-26 = Eating Attitudes Test-26 (Garner, Olmstead, Bohr, & Garﬁnkel, 1982), EDE = The Eating Disorders Examination (Fairburn & Cooper, 1993), EDE-Q = Eating Disorder Examination Questionnaire (Fairburn & Beglin, 1994), EDI = The Eating Disorder Inventory (Garner, 2004), EDI-2 = The Eating Disorder Inventory–2 (Garner, 1991), ER = Emotions Rating (Vocks et al., 2007), ESS = Experience of Shame Scale (Andrews, Qian, & Valentine, 2002), FbeK = Fragebogen zur Beurteilung des eigenen Körpers *[Questionnaire for evaluations of one’s own body*] (Strauß & Richter-Appelt, 1996), HC = healthy controls, MBSRQ = Multidimensional Body-Self Relations Questionnaire (Brown, Cash, & Mikulka, 1990), MtF GID = Male-to-female Gender Identity Disorder, NSSI = non-suicidal self-injury, OBC = Objectiﬁed Body Consciousness Scale (McKinley & Hyde, 1996), RFQ = Risky Family Questionnaire (Taylor, Lerner, Sage, Lehman, & Seeman, 2004), SBA = Survey of Body Areas (Kleindienst et al., 2014), TC = Thoughts Checklist (Cooper & Fairburn, 1992), TEC = The Dutch Traumatic Experiences Checklist (Nijenhuis et al., 2002), The Childhood Sexual Abuse Measure (Finkelhor, 1979)

Table S2

Quality Assessment of studies included in the systematic review

|  | 1.Research Question | 2.Study population | 3.Participation rate | 4.Recruit-ment | 5. Power | 6.Categories of exposure | 7.Exposure measures | 8.Outcome measures | 9.Confounders | Total scores | Rating |
| --- | --- | --- | --- | --- | --- | --- | --- | --- | --- | --- | --- |
| Andrews (1995) | 1 | 0,5 | NR | 1 | 0 | 0 | CD | 0,5 | 0,5 | 3,5 | poor |
| Bandini et al. (2011) | 1 | 1 | 1 | 0,5 | 0 | 0 | CD | 1 | 0,5 | 5 | poor |
| Bhandari et al. (2011) | 1 | 1 | 0 | 0 | 1 | 0,5 | CD | 1 | 0,5 | 5 | poor |
| Borgmann et al. (2014) | 1 | 1 | 1 | 0 | 0 | 0,5 | 1 | 1 | 1 | 6,5 | fair |
| Brooke & Mussap (2013) | 1 | 1 | NA | 1 | 0 | 1 | 1 | 1 | 1 | 7 | good |
| Dunkley et al. (2010) | 1 | 0,5 | NA | CD | 0 | 1 | 1 | 1 | 0,5 | 5 | poor |
| Dyer et al. (2013) (1) | 1 | 1 | 0 | 0,5 | 0 | 0,5 | 1 | 1 | 1 | 6 | fair |
| Dyer et al. (2013) (2) | 1 | 1 | NR | 0,5 | 0 | 0,5 | 1 | 1 | 0,5 | 5,5 | fair |
| Dyer et al. (2015) | 1 | 1 | NR | 0 | 0 | 0,5 | 1 | 1 | 0,5 | 5 | poor |
| Eubanks et al. (2006) | 1 | 1 | NA | 1 | 0 | 0,5 | 1 | 1 | 0,5 | 6 | fair |
| Grilo & Masheb (2001) | 1 | 1 | NR | 0,5 | 0 | 1 | 1 | 1 | 0,5 | 6 | fair |
| Grilo et al. (2005) (1) | 1 | 1 | NR | 0,5 | 0 | 0,5 | 1 | 1 | 0,5 | 5,5 | fair |
| Grilo et al. (2005) (2) | 1 | 1 | NR | 0,5 | 0 | 1 | 1 | 1 | 0,5 | 6 | fair |
| Grilo et al. (2006) | 1 | 1 | NR | 1 | 0 | 1 | 1 | 1 | 0,5 | 6,5 | fair |
| Hund & Espelage (2006) | 1 | 1 | NA | 1 | 0 | 0,5 | 1 | 1 | 1 | 6,5 | fair |
| Hunter (1991) | 1 | 1 | NA | 1 | 0 | 0,5 | 0,5 | 1 | 0,5 | 5,5 | poor |
| Jenkins et al (2013) | 1 | 1 | NA | 1 | 0 | 1 | 1 | 1 | 0,5 | 6,5 | fair |
| Kilimnik & Meston (2016) | 1 | 1 | 1 | 1 | 0 | 0,5 | 1 | 1 | 0,5 | 7 | good |
| Mahtani et al. (2019). | 1 | 1 | NA | 1 | 0,5 | 1 | 0,5 | 1 | 0,5 | 6,5 | fair |
| Maseroli et al. (2018) | 1 | 1 | NR | 0,5 | 0 | 0,5 | 1 | 1 | 1 | 6 | fair |
| Milligan & Andrews (2005) | 1 | 1 | 1 | 1 | 0 | 0,5 | CD | 1 | 0,5 | 6 | fair |
| Möller et al. (2018) | 1 | 1 | NR | 1 | 0 | 1 | 1 | 1 | 0 | 6 | fair |
| Muehlenkamp et al. (2011) | 1 | 1 | 1 | 1 | 0 | 1 | 1 | 1 | 0,5 | 7,5 | good |
| Nagaraj et al. (2019) | 1 | 1 | NA | 0,5 | 0 | 0,5 | 0,5 | 1 | 0,5 | 5 | poor |
| Preti et al. (2006) | 1 | 0,5 | 0,5 | 1 | 0,5 | 0 | 0 | 1 | 1 | 5,5 | fair |
| Rohde et al. (2008) | 1 | 1 | 1 | 1 | 0,5 | 0,5 | 0 | 0 | 0,5 | 5,5 | fair |
| Salmon et al. (2006) | 1 | 0,5 | 1 | 1 | 0 | 1 | CD | 1 | 0,5 | 6 | fair |
| Schaaf & McCanne (1994) | 1 | 1 | NR | 1 | 0 | 0,5 | 1 | 1 | 1 | 6,5 | fair |
| Scheffers et al. (2017) | 1 | 1 | 1 | 0,5 | 0 | 0,5 | CD | 1 | 0,5 | 5,5 | fair |
| Senior et al. (2005) | 1 | 1 | NR | 1 | 0 | 0,5 | CD | 0,5 | 0,5 | 4,5 | poor |
| Talmon & Ginzburg (2018) | 1 | 1 | NA | 1 | 0 | 1 | 1 | 1 | 0,5 | 6,5 | fair |
| Talmon & Ginzburg (2019) | 1 | 1 | NA | 1 | 0 | 1 | 1 | 1 | 0,5 | 6,5 | fair |
| Treuer et al. (2005) | 0,5 | 0,5 | NR | 1 | 0 | 0,5 | CD | 1 | 0,5 | 4 | poor |
| Vartanian et al. (2018) | 1 | 1 | 1 | 1 | 0 | 1 | 1 | 1 | 1 | 8 | good |
| Walsh et al. (2017) | 1 | 1 | 1 | 1 | 0 | 0,5 | 1 | 1 | 1 | 7,5 | good |
| Watson et al. (2013) | 1 | 1 | NR | 1 | 1 | 0,5 | 1 | 1 | 0,5 | 7 | good |
| Wenninger & Heiman (1998) | 1 | 1 | 1 | 1 | 0 | 0,5 | CD | 1 | 1 | 6,5 | fair |
| Wonderlich et al. (1996) | 1 | 1 | 1 | 1 | 0 | 0,5 | 1 | 1 | 1 | 7,5 | good |
| Wonderlich et al. (2001) | 1 | 1 | NR | 0.5 | 0 | 0,5 | CD | 1 | 0,5 | 4 | poor |
| Zlotnick et al. (1996) | 1 | 1 | NR | 1 | 0 | 0,5 | CD | 1 | 0,5 | 5 | poor |
| *Note*. CD: Cannot Determine; NA: Not Applicable; NR: Not Reported. 1. Was the research question or objective in this paper clearly stated? 2. Was the study population clearly specified and defined? 3. Was the participation rate of eligible persons at least 50 %? 4. Were all subjects selected or recruited from the same or similar populations? Were inclusion and exclusion criteria for being in the study prespecified and applied uniformly to all participants? 5. Was a sample size justification, power description, or variance and effect estimates provided? 6. For exposures that can vary in amount or level, did the study examine different levels of the exposure as related to the outcome (e.g., categories of exposure, or exposure measured as continuous variable)? 7. Were the exposure measures (independent variables) clearly defined, valid, reliable, and implemented consistently across all study participants? 8. Were the exposure outcome measures clearly defined, valid, reliable and implemented consistently across all study participants? 9. Were key potential confounding variables measured and adjusted statistically for their impact on the relationship between exposure and outcome? | | | | | | | | | | | |
